# Neurodegenerative diseases: a hotbed for splicing defects and the potential therapies

**DOI:** 10.1186/s40035-021-00240-7

**Published:** 2021-05-20

**Authors:** Dunhui Li, Craig Stewart McIntosh, Frank Louis Mastaglia, Steve Donald Wilton, May Thandar Aung-Htut

**Affiliations:** 1 Centre for Molecular Medicine and Innovative Therapeutics, Health Futures Institute, Murdoch University, Perth, Western Australia Australia; 2 Perron Institute for Neurological and Translational Science, Centre for Neuromuscular and Neurological Disorders, The University of Western Australia, Perth, Western Australia Australia

**Keywords:** Neurodegenerative diseases, Parkinson’s disease, Alzheimer’s disease, Alternative splicing, Splicing defects, Antisense oligonucleotides, Splice-switching, Disease-modifying treatment

## Abstract

Precursor messenger RNA (pre-mRNA) splicing is a fundamental step in eukaryotic gene expression that systematically removes non-coding regions (introns) and ligates coding regions (exons) into a continuous message (mature mRNA). This process is highly regulated and can be highly flexible through a process known as alternative splicing, which allows for several transcripts to arise from a single gene, thereby greatly increasing genetic plasticity and the diversity of proteome. Alternative splicing is particularly prevalent in neuronal cells, where the splicing patterns are continuously changing to maintain cellular homeostasis and promote neurogenesis, migration and synaptic function. The continuous changes in splicing patterns and a high demand on many *cis-* and *trans-*splicing factors contribute to the susceptibility of neuronal tissues to splicing defects. The resultant neurodegenerative diseases are a large group of disorders defined by a gradual loss of neurons and a progressive impairment in neuronal function. Several of the most common neurodegenerative diseases involve some form of splicing defect(s), such as Alzheimer’s disease, Parkinson’s disease and spinal muscular atrophy. Our growing understanding of RNA splicing has led to the explosion of research in the field of splice-switching antisense oligonucleotide therapeutics. Here we review our current understanding of the effects alternative splicing has on neuronal differentiation, neuronal migration, synaptic maturation and regulation, as well as the impact on neurodegenerative diseases. We will also review the current landscape of splice-switching antisense oligonucleotides as a therapeutic strategy for a number of common neurodegenerative disorders.

## Introduction

Neurodegenerative diseases are a large and heterogenous class of disorders that are categorised primarily by the loss of function or structural integrity of neurons and associated cell types in the nervous system. Typically, these diseases are progressive in nature and often, but not always, manifest in adult life, with the vast majority of diseases having no cure or effective treatment strategy [1–5]. The progressive loss of functional neurons underlies the cognitive and motor impairments seen in neurodegenerative diseases [6]. The most common pathological feature observed in neurodegenerative diseases is the accumulation of insoluble misfolded protein aggregates [7–10]. These aggregates take various constitutive forms, depending on the specific disease type and/or genetic cause. Most cases of neurodegeneration are sporadic, but common genetic forms can be caused by mutations in the gene that lead to conformational changes of the encoded protein, making the protein highly likely to misfold and aggregate [3]. Although neurodegenerative diseases are mainly sporadic, there are clear underlying genetic causes for neurodegenerative diseases, and errors that affect normal splicing are relatively common [4].

At the completion of the Human Genome Project, it was determined that there are approximately 23,000 protein-coding genes in the human genome. Interestingly, the number of genes has no relation to the complexity of an organism, as the common wheat plant carries roughly 95,000 protein-coding genes, while the loblolly pine tree contains a genome (23 billion bases) that is roughly 10 times that of the human (2.3 billion bases) [11, 12]. It has been established that alternative splicing is responsible for the great discrepancy among the ~23,000 protein-coding genes in the human genome, which gives rise to ~200,000 different gene transcripts and around 2 million different proteins that they encode [13]. Alternative splicing is a process whereby multiple different mRNA isoforms arise from a single protein-coding gene, achieved by the exclusion or inclusion of single or multiple exons, or by the use of alternative splicing sites to give rise to partial exons or the retention of an intronic sequence, in essence blurring the strict definition of exons and introns unless temporal, spatial, environmental or tissue-specific caveats are taken into consideration [14]. However, to fully understand the mechanisms that lead to alternative splicing and thus potential disease-causing splicing mutations, one must first understand the process of precursor messenger RNA (pre-mRNA) splicing as a whole.

### Pre-mRNA splicing

**Fig. 1:**
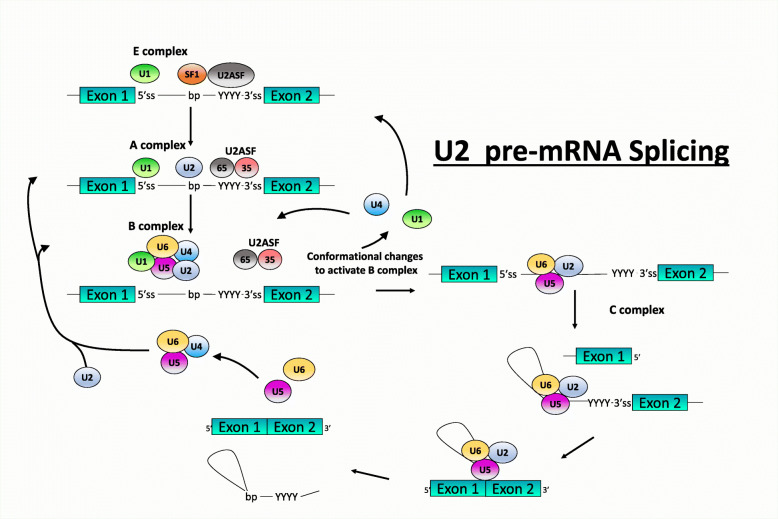
Schematic of the process of pre-mRNA splicing and major spliceosome assembly. Initial assembly into Complex E involves binding of the U1 snRNP (U1) to the 5’splice site (ss), while non-snRNP splicing factor 1 (SF1) and U2AF bind to the branchpoint sequence and polypyrimidine tract, respectively [16]. Subsequently, U2 snRNP is recruited by SF1 and U2AF, replaces SF1 to bind to the branchpoint, and initiates the formation of Complex A. The recruitment of U2 then facilitates enlistment of the U4/U6-U5 tri-snRNP that is pre-assembled from the U4/U6 and U5 snRNPs, thus forming the pre-catalytic Complex B. Next, destabilisation of U4 and U1 leads to the dissociation of U4, while U6 replaces U1 at the 5’ss and gives rise to the activated spliceosome. This catalytically activated Complex B initiates the first step in splicing, giving rise to Complex C that then cleaves the 5’ss, releasing the first exon to fold and the 5’ss can now join to the branchpoint, forming a lariat within the intron. Following the lariat formation is the second step in splicing; cleavage of the intron at the 3’ss, release of the lariat and the ligation of the two bordering exons. Upon completion of the final step, the spliceosome dissociates so that the snRNPs may be recycled and splicing of a subsequent intron occurs. This is repeated until all the introns from the mRNA are removed, thus giving rise to the formation of the mature mRNA transcript [17, 18]. Following intron excision and ligation of the exons, the U snRNPs are recycled. 5’ss, 3’ss, bp and polypyrimidine tracts are shown in the line representing the intron. Exons are shown as magenta boxes. Adapted from Pitout (2019) [19].

Pre-mRNA splicing is an integral step in “split” gene expression, which occurs in all higher eukaryotes and some lower eukaryotes. All pre-mRNA transcripts contain defined sequences destined for inclusion in the mature mRNA isoform (exons) and are separated by sequences that are excluded from the mature mRNA (introns) [15]. During mRNA maturation, the introns are removed whilst the exons are assembled and precisely ligated together to form a continuous mature mRNA transcript, ready for export and potential protein translation or regulatory function. The splicing processing requires a highly coordinated arrangement of numerous splicing RNA and protein factors that act together with a range of splicing motifs to produce a large multi-protein complex termed the spliceosome to coordinate these molecular gymnastics (Fig. 1) [20]. Considered most simplistically, this process consists of two sequential transesterification reactions that ligate adjacent exons. However, this process is far from simple and involves hundreds of interacting proteins and small nuclear RNAs (snRNAs) and a number of small nuclear ribonucleoproteins (snRNPs). In the absence of mutations, the process of splicing is highly ordered and precise, involving several multi-component splicing factors with the addition of the above mentioned snRNPs (Fig. 1). Pre-mRNA splicing is so finely balanced and intricately tuned that errors in *cis-* and/or *trans-*splicing motifs/factors can commonly occur and are thought to account for up to a third of all human diseases [21], in particular neurodegenerative diseases.

### Alternative Splicing

**Fig. 2:**
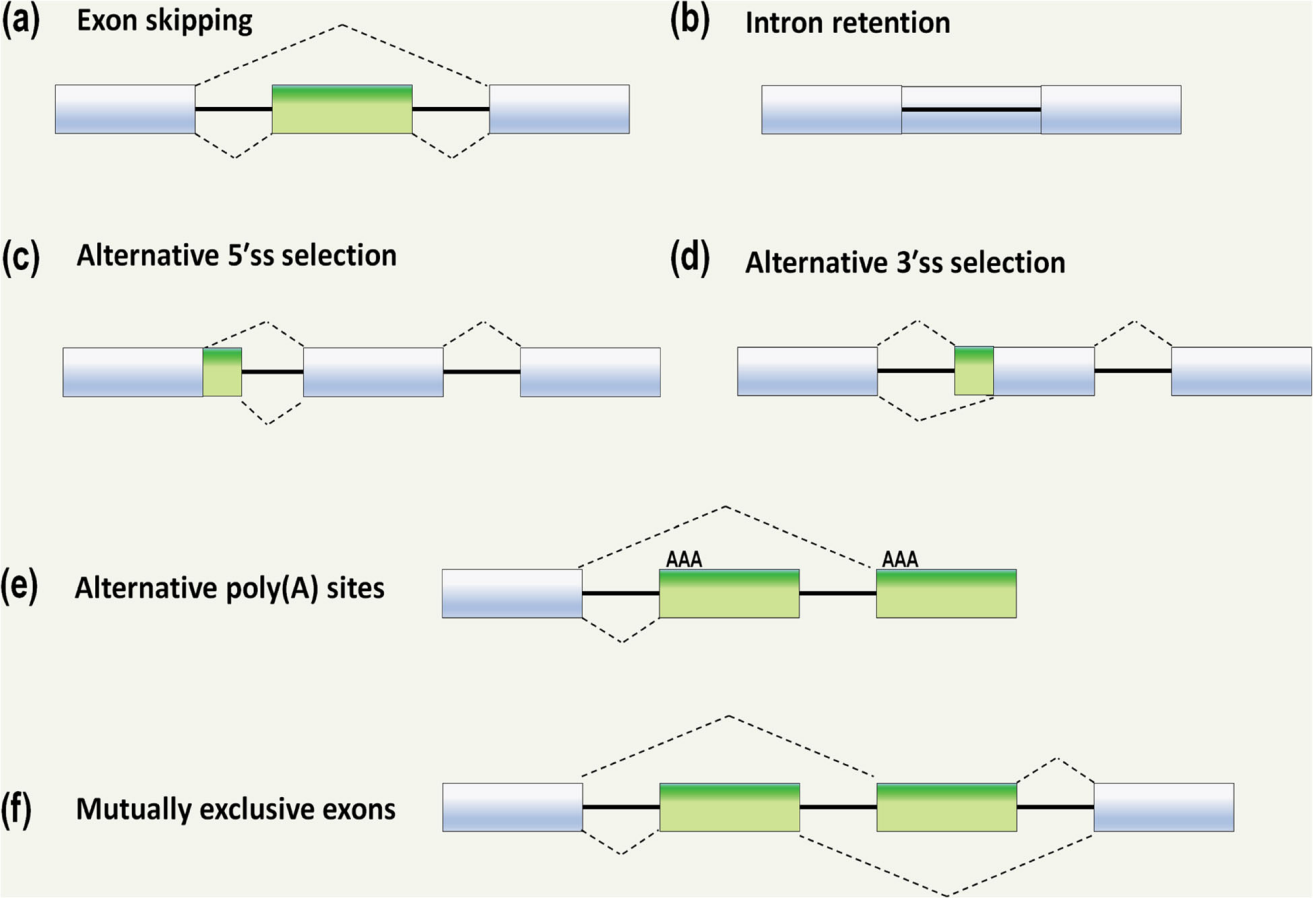
Schematic of the most common forms of alternative splicing. **a** Exon skipping. **b** Intron retention. **c** Alternative 5’ splice site (ss) selection. **d** Alternative 3’ ss selection. **e** Alternative polyadenylation (polyA) sites. **f** Mutually exclusive exons. Light blue boxes denote segments included in the final message, while green boxes denote segments excluded in the mature mRNA transcript. Dotted lines show the splicing pattern. Note: mechanisms are not mutually exclusive, and combinations can often occur.

As previously mentioned, alternative splicing is critical not only for the diversification in specific species, but also for tissue specificity within organisms. Furthermore, the differences in splicing and ultimately mRNA sequence may have an effect on mRNA stability, localisation and translation [22], resulting in various protein isoforms with distinct and sometimes opposing biological functions. The common mechanisms of alternative splicing are shown in Fig. 2. A perfect example of this is the alternative splicing within the receptor for advanced glycosylation end products (*RAGE*) gene. RAGE is a multiligand receptor of the immunoglobulin superfamily that plays an integral role in inflammation and innate immune response. The alternative splicing of *RAGE* leads to three main isoforms with distinct biological functions, the full-length membrane RAGE (mRAGE), soluble RAGE (sRAGE) and N-truncated RAGE (NtRAGE) [23–25].

There are numerous *cis-*acting elements that regulate splicing and it is these elements that may cause subtle differences in recognition of the exon by the spliceosome, giving rise to alternatively spliced transcripts [14]. Alternative exons or sequences share similar 3’ and 5’ splice sites; however, they typically have a weaker binding affinity to the spliceosome than consensus exons, resulting in reduced recognition [26]. Next to splice site recognition, splicing factors play a major role in alternative exon recognition. Serine and arginine-rich (SR) proteins typically enhance the recognition of alternative exons, while heterogeneous nuclear ribonucleoproteins (hnRNPs) conversely aid in exclusion of the exon from the mature mRNA transcript. However, as in many cases in biology, there are clear exceptions to these generalisations [27, 28], where two SR proteins, SF2/ASF and hTra2-beta, have been shown to cause skipping of several ceramide-regulated exons from the mature mRNA isoforms [27].

Alternative splicing is evidently an integral component of the neuronal gene expression network that regulates cell differentiation, tissue homeostasis and organ development [22]. A key feature is the tissue-specific alternative splicing, whereby specific mRNA isoforms from the same gene are selectively expressed and translated in different tissues or cell types or during specific stages of development or metabolic conditions. However, with a high degree of diversity and complexity, there is an increased potential for splicing errors, and in fact, errors in alternative splicing and in splicing in general are known to play many roles in diseases [15, 29–31].

## Tissue-specific splicing in the brain

Alternative splicing is a fundamental aspect during the complex life cycle of a neuron, which occurs constantly throughout early neuronal differentiation to synapse formation, supporting cell plasticity and signalling, and is even critical for programmed cell death [32–35]. This extraordinarily complex and coordinated phenomenon creates a plethora of splice isoforms across various neuronal cell-types during development and adaptation [36, 37]. The nervous system is a well-established hotbed for alternative splicing of pre-mRNAs, which has been clearly shown to be a central mechanism underlying many neuronal functions [38–42]. Additionally, numerous splice sites seem to be evolutionarily conserved, which is consistent with a view that alternative splicing plays a central role in encoding properties essential for neuronal functions [43–46]. In fact, alternative splicing could be considered the norm in neuronal gene expression, rather than the exception, with the fact that between 15%–50% of human genetic diseases arise from mutations affecting the alternative splicing processes [47].

### Alternative splicing and neuronal differentiation

The brain is an extremely complex organ with numerous cell types working in coordination to achieve homeostasis. In order to produce this intricate mesh of cell types there is a requirement for organised and coordinated activation and inactivation of transcriptional regulators. In addition, there is delicate and coordinated expression of various *trans-*acting splicing factors that bind *cis-*elements in pre-mRNAs to either promote or hinder recruitment of the spliceosome at intron/exon boundaries (Fig. 1) [26, 48]. Splicing factors bind to single or clusters of RNA motifs located in introns and exons, to either enhance or inhibit target exon inclusion as required [49]. In a neurological setting, specific RNA-binding proteins, particularly polypyrimidine tract binding protein 1 (PTBP1) and SR-rich (serine/arginine) repetitive matrix protein 4 (SRRM4), play an essential role in cellular differentiation [33, 50–52]. It is now known that the alternative splicing patterns of *PTBP1* and *SRRM4* transcripts undergo drastic changes over the course of early neurogenesis [53].

#### PTBP1

Members of the PTB family share a high degree of homology and function but nevertheless exhibit distinct cell-type expression patterns. For example, the full-length *PTBP1* is largely absent in mature tissues such as neurons and muscles, while in tissues such as neuronal progenitors and neuronal stem cells, *PTBP1* is highly expressed [54]. The expression of *PTBP1* is known to decrease sharply upon mitotic exit (or maturation) through mRNA downregulation by the neuron-specific microRNA, miR-124 [55, 56]. Mechanistically, miR-124 has been shown to target *PTBP1* mRNA directly, and this downregulation of PTBP1 globally represses non-neuronal alternative pre-mRNA splicing [55]. Among the binding targets of *PTBP1* is a key cassette exon located within the pre-mRNA of PTB family member, *PTBP2* [57].

*PTBP1* is highly expressed in the early stages of neurogenesis, which in turn promotes PTBP1 binding to *PTBP2*, subsequently causing the cassette exon to be skipped, thereby subjecting *PTBP2* to nonsense-mediated decay [55]. However, during the late stage of neuronal differentiation, high levels of miR-124 repress *PTBP1* expression (thus reducing PTBP1 binding to *PTBP2*), leading to the inclusion of the cassette exon located in *PTBP2* and thus an increase in the full-length *PTBP2* transcript. This subtle difference in *PTBP1* expression induces the transition of alternative splicing from a non-neuronal to a neuronal-specific pattern [55]. Another critical gene that interacts with PTBP1 for neuronal differentiation is *SRRM4*.

#### SRRM4

SRRM4 is similar in structure to the serine/arginine (SR)-rich splicing factor family, and is a crucial factor for alternative splicing patterns found exclusively in neuronal cells [58]. Although SRRM4 lacks typical RNA-binding domains, it commonly binds to UGC-rich motifs that are located between the 3’-splice site and the polypyrimidine tract [22]. The most common motifs targeted by SRRM4 are typical PTBP1-binding targets, leading to the notion that SRRM4 antagonises PTBP1. SRRM4 participates in neurogenesis through its role in neurite outgrowth [59]. In 2016, Nakayama and colleagues demonstrated that SRRM4 regulates splicing of protrudin gene (*Zfyve27*) transcripts in mouse Neuro2A cells. They showed that SRRM4 promotes the inclusion of a micro-exon (encoding only seven amino acids) within the mature transcript of protrudin, through the UGC-rich motif that is located immediately upstream of the micro-exon [59]. The resulting protein, termed protrudin-L, was shown to promote neurite outgrowth during neurogenesis. In fact, SRRM4 has broad effects on the selection of neuronal specific micro-exons [58]. Several SRRM4-regulated micro-exons have demonstrated high levels of inclusion during neuronal maturation, which is directly correlated to the high levels of *SRRM4* [58, 60, 61]. Following neuronal differentiation, maturation and synaptogenesis occur over a sustained period of time.

### Alternative splicing and neuronal migration

Following differentiation, neuronal cells need to migrate to their respective brain regions, and similar to differentiation, this process relies heavily on alternative splicing, particular the alternative splicing factor neuro-oncological ventral antigen 2 (NOVA2).

NOVA2 protein has been shown to regulate transcripts that encode synapse-related molecules in the postnatal brain as well as playing a critical role in neuronal cell migration. There are two NOVA paralogues, NOVA1 and NOVA2; they both contain KH-type RNA-binding domains. NOVA1 is primarily expressed in the ventral spinal cord and hindbrain, while NOVA2 is expressed in the dorsal spinal cord and forebrain [62]. Their critical involvement in neuronal migration and differentiation is evident with severe phenotypes observed in knockout models [62]. The *NOVA2* gene is critical for proper cortical lamination. In *Nova2* knockout mice, neuronal migration defects occurred in both the cerebral cortex and the cerebellum [63], while the progenitor cell morphology was mostly unaffected. This suggests a defect in neuronal migration rather than complications arising from incorrect tissue subtype specification [63]. The defective migration of the upper layers of mouse neurons is widely attributed to the mis-splicing of disabled 1 (*Dab1*) [64].

Dab1 is a protein involved in the Reelin signalling pathway, a pathway that is responsible for the positioning of neurons, as well as the growth, maturation and synaptic activity of neuronal cells [65, 66]. In the presence of NOVA2, two exons (exons 7b and 7c) found within the *Dab1* transcript are excluded from the mature mRNA, resulting in an unstable Dab1 isoform that is tagged for ubiquitination upon activation of the Reelin pathway [63, 67]. Conversely, in the absence of NOVA2, these exons are included and provide stability to the specific Dab1 isoform [63, 67]. The role of NOVA2 is not limited to neuronal migration, but also in synaptic maturation and plasticity. This suggests that NOVA proteins are critical to brain-specific splicing through multiple stages of development.

### Alternative splicing and synaptic maturation and regulation

Once the cell fate and the location are determined, a high degree of alternative splicing is still needed for neuronal cells to undergo maturation and function properly. Genes such as *PTBP1*, *PTBP2*, *SRRM4*, *NOVA2*, and RNA binding Fox-1 Homolog 1/2 (*RBFOX1/2*) play a critical role in the maturation and on-going functionality [14, 22, 44, 68].

#### Synaptic maturation

Compared to most other cell types, neurons undergo an unusually long maturation period. Once signalling for differentiation and migration comes to an end, changes in splicing patterns lead to the development from initial neurites to defined axons and dendrites, which finally assemble to form active synaptic connections and signalling [57, 69]. One of the initial changes observed is a dramatic reduction in the level of splicing factor PTBP1, while the level of the related PTBP2 protein increases. The shift in expression denotes the end of cell differentiation and the commencement of maturation. Homozygous knockout of *Ptbp2* in mice leads to the degeneration of cortical neurons during a developmental period which otherwise should see the cortex expand and develop mature working synapses [69]. Although PTBP2 depletion leads to degeneration, it does not hinder neuronal differentiation, suggesting that PTBP2 is not necessary during early neurogenesis [69]. Mechanistically, the depletion of PTBP2 can cause mis-regulations of several splicing patterns in the mouse brain, with the PTBP2-targeted exons/transcripts known to encode proteins that affect neurite growth and synaptic transmission and assembly [69]. Apart from PTBP2, SRRM4 has also been shown to be an integral factor involved in synaptogenesis.

Several targets of SRRM4 overlap with those of PTBP2, suggesting a similar role of them in synaptic maturation. In fact, *Srrm4*^*Δ7-8*^ mice exhibit a similar phenotype to the previously described *Ptbp2* knockout model [58]. The *Srrm4*^*Δ7-8*^ mice carry a heterozygous conditional deletion of exon 7 and exon 8 throughout the animal and in the germline, resulting in widespread loss of the functional full-length protein [58]. This loss of functional Srrm4 leads to aberrant splicing in the brain in several gene transcripts implicated in vesicle trafficking. Homozygous *Srrm4*^*Δ7-8/Δ7-8*^ mice display a severe phenotype with 85% mortality within the first few weeks of life. Although the mice show no obvious gross morphological phenotype, they soon develop respiratory complications and cyanosis [58]. Interestingly, the surviving mice did not show a significant difference in life span when compared to the wild-type littermates but displayed pronounced neurobiological phenotypes. These findings suggest that Srrm4 plays a role in developmental neurogenesis and in normal synaptic functioning.

#### Synaptic regulation

Once synapses are fully formed, the regulation and function of each synapse still requires high levels of alternatively spliced genes. At the forefront of this are the splicing factors RBFOX1 and RBFOX2 [33, 70]. It has been shown that mutations in *RBFOX1* lead to various epileptic phenotypes, indicating its role in synaptic excitability. Transcriptome analysis of homozygous *Rbfox1*^*-/-*^ mouse brains showed numerous splicing changes in target transcripts of Rbfox1, although no significant change in transcript abundance was observed [70]. These changes in splicing pattern were shown to affect proteins that mediate synaptic excitation and transmission. The phenotype of the mice seemed to confirm this finding as they had spontaneous, infrequent seizures when compared to wild-type mice [70]. Complementary to this, Jacko et al. (2018) generated triple *Rbfox1/2/3* knockout (tKO) spinal neurons to examine and characterise the complex network of alternatively spliced genes targeted by the Rbfox family [33]. The tKO neurons harboured several alternative splicing defects in genes encoding proteins responsible for the regulation of neuronal membranes and synaptic function [33]. In fact, tKO neurons appear to display immature electrophysiological activity, through diminished axon initial segments, a structure critical for action potential initiation [33]. The tKO neurons were shown to have more severe splicing changes when compared to murine brains in which individual *Rbfox* genes were knocked out, highlighting important roles of all three *Rbfox* genes in the regulation of alternative splicing in the brain [33, 70–72].

It is clear that the brain is a hotbed for alternative splicing, and although alternative splicing is invaluable, it does come with several potential drawbacks. Many neurodegenerative diseases have been linked to defects in splicing, including Parkinson’s disease (PD), Alzheimer’s disease (AD) and spinal muscular atrophy (SMA).

## Alternative splicing and splicing defects in neurodegenerative diseases

### PD

PD is a progressive neurodegenerative disorder whose aetiology is thought to involve an interaction between a wide range of environmental toxins and genetic risk factors. PD has a pathological hallmark of the presence of intraneuronal cytoplasmic inclusions, called Lewy bodies. The major component of Lewy bodies is alpha-synuclein (SNCA), which is a 14 kDa protein that regulates synaptic vesicle release and trafficking, membrane channel formation, and neurotransmitter release [73]. Mutations in *SNCA*, including missense mutations A53T and A30P or overexpression (through duplication or triplication of the *SNCA* gene), cause SNCA misfolding and an increase in the relative expression of SNCA, thereby promoting SNCA oligomerization and aggregation. As SNCA aggregates, fibrils eventually impair many molecular pathways including autophagy, the ubiquitin-proteasome protein degradation system, and mitochondrial homeostasis [74–76]. In addition, emerging evidence shows that different SNCA isoforms, generated from *SNCA* alternative splicing, have different aggregation propensities and thus play an important role in PD pathophysiology.

Multiple minor *SNCA* transcripts have been reported, and although not prevalent, these transcripts are primarily alternatively spliced at the 5’-untranslated region (UTR) or 3’-UTR. For the 5’-UTR, over 10 different initial exons have been reported, with varying lengths [77]. With respect to the 3’-UTR, brain-specific alternative selection of polyadenylation sites generates at least three different *SNCA* transcripts with varying lengths of 3’-UTR. However, the differences in the length of 5’-UTR and 3’-UTR have been suggested to have no impact on the overall total protein production or the coding sequence [78].

**Fig. 3.**
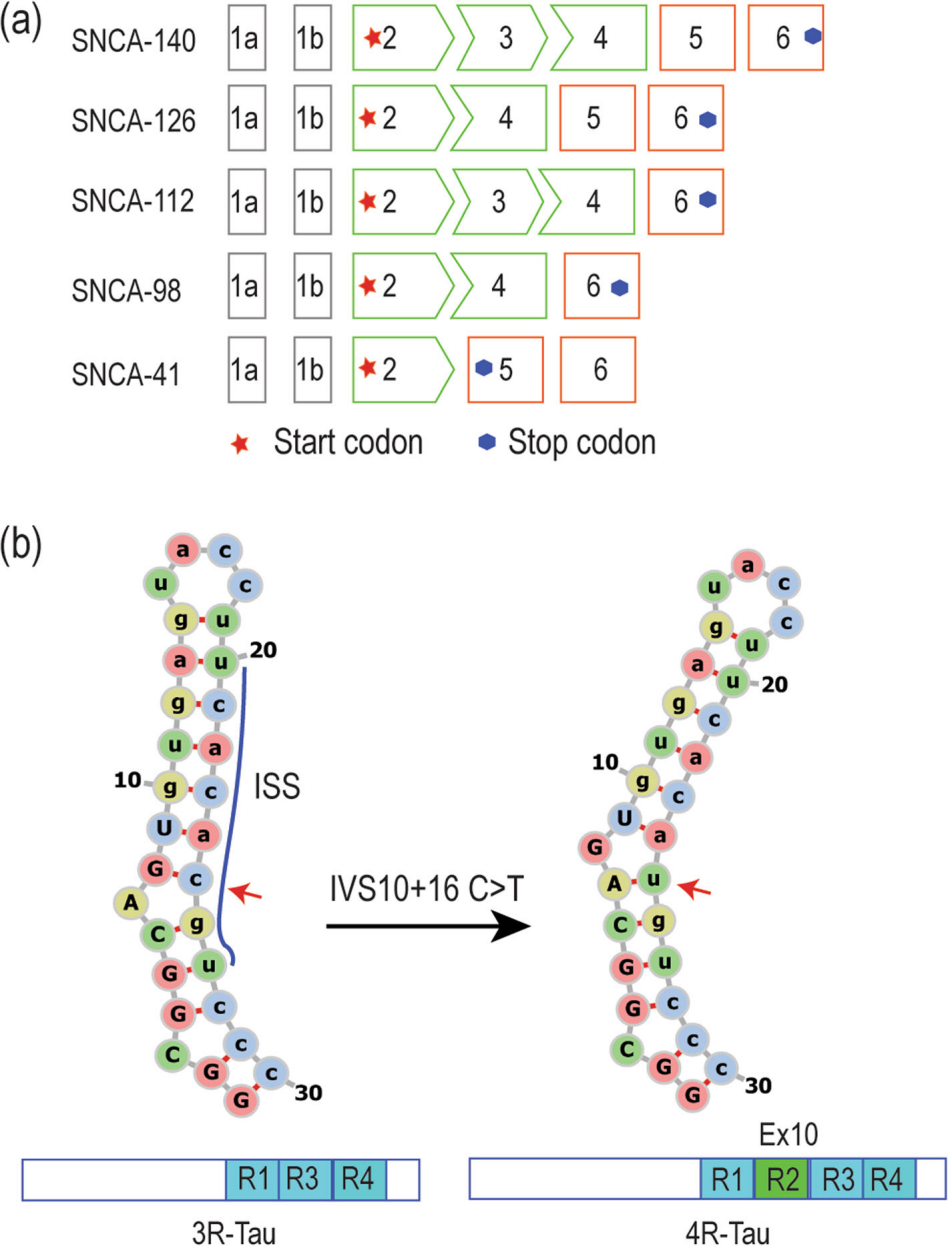
Alternative transcripts of *SNCA* and *MAPT*, and the stem loop near *MAPT* exon 10 donor splice site. **a** Five *SNCA* alternative transcripts resulting from skipping of exon(s) 3, 4, and/or 5. **b** Tau isoforms with three (3R) or four (4R) C-terminal microtubule binding repeats due to alternative splicing of *MAPT* exon 10. Self-complementary stem loop at the 3’-end of exon 10 and the 5’-end of intron 10 and a strong intron splicing silencer (ISS) interfere with the pairing of U1 small nuclear RNA to *MAPT* exon 10, weakening exon 10 inclusion. The intronic mutation IVS10+16 C>T (as indicated by arrows) disrupts the ISS encoded by sequence 5’-ucacacgu-3’ and increases *MAPT* exon 10 inclusion. Exonic sequences are shown in capital letters; intronic sequences are in lower cases. Ex: exon; R: repeat.

There are five main *SNCA* transcripts resulting from alternative splicing of *SNCA* exon 3, exon 5, or both (Fig. 3a). The full-length *SNCA* (*SNCA140*) is the most abundant transcript, making up 96.7%–98.1% of the total *SNCA* mRNA transcripts [79]. The expression levels of these alternatively spliced isoforms are very low in healthy populations, but vary in patients with PD, diffuse Lewy body dementia (DLBD) and MSA [80]. The *SNCA112* transcript arises from the removal of exon 5, resulting in deletion of 28 amino acids at the C-terminal of the SNCA protein. The loss of three glutamic acids and an aspartic acid increases the net charge of SNCA, thus making SNCA112 more prone to aggregation compared to the full-length isoform. In addition, splicing out exon 5 results in the loss of amino acid S129, which is the major phosphorylation site of SNCA and is involved in SNCA clearance, aggregation and toxicity [81]. The loss of S129 has been suggested as the determinant factor for the higher aggregation properties of SNCA112 compared to SNCA140.

In contrast, the *SNCA126* transcript that lacks exon 3 encodes a protein that shows low aggregation rates due to the loss of the highly amyloidogenic non-amyloid component region that contributes to SNCA oligomerization and aggregation. Since the C-terminal is unaffected in the SNCA126 isoform, the net charge of this isoform is even lower than SNCA140, thus SNCA126 is less likely to aggregate compared to the SNCA112 and SNCA140 isoforms. Clinical observations have demonstrated that SNCA126 is diminished in the frontal cortex of DLBD patients at synucleopathogy stages 5 and 6 [82]. However, PD patients at stages 3 and 4 show high levels of SNCA126, suggesting that SNCA126 may have some protective potential against the latter stages of disease. The *SNCA41* transcript is a newly identified *SNCA* alternative transcript expressed in PD brains [83]. The skipping of exons 3 and 4 generates a 238-bp transcript with a premature termination codon, which is translated into a short SNCA isoform of 41 amino acids. SNCA41 neither aggregates nor affects the fibrillation of full-length SNCA and is not deleterious to dopaminergic cells *in vitro*. However, PC12 cells pre-treated with recombinant SNCA41 showed increased dopamine uptake [83]. Since different SNCA isoforms have various pathophysiological functions, understanding the underlying mechanisms of the differential expression of these isoforms could provide insights into the development of novel therapeutic strategies.

Single nucleotide polymorphisms (SNPs) are natural genetic variants and can have neutral, functional or catastrophic biological effects [84]. The role of SNPs in PD pathogenesis is unequivocal, with several SNPs in the 3’-UTR being shown to alter the *SNCA* enhancer activity and lead to overexpression of *SNCA* [85, 86]. SNPs are also suggested to affect the expression of different SNCA isoforms. For example, SNP in intron 4 (rs2736990) and SNPs in 3’-UTR (rs356165 and rs356219) are associated with an increase in *SNCA112* expression [87, 88]. As potential *cis-*acting splicing motifs are found around the sequences surrounding the SNPs, those SNPs are suggested to disrupt the splicing context and redirect normal *SNCA* splicing [89]. As *SNCA* structural variants, polymorphic microsatellites have been found to contribute to synucleinopathies through regulating *SNCA* gene expression and splicing [90]. The splicing efficiency of *SNCA* exon 3 is associated with one of three poly(T)*n* variants in *SNCA* intron 2: the 5T-allele, 7T-allele, and 12T allele [91]. Higher expression levels of *SNCA126* are correlated with longer polyT stretch in the normal brain [77].

Alternative splicing events that affect cellular functions of proteins have also been found in other genes related with PD, and aberrant splicing in these genes is suggested to contribute to the PD pathogenesis. Mutations in leucine-rich repeat kinase 2 (*LRRK2*) are the most common cause for the sporadic and late-onset familial PD [92]. *LRRK2* functions are mainly affected by missense mutations spreading across the gene; however, several pathogenic mutations have been reported to affect the *LRRK2* splicing [93]. Homozygous or compound heterozygous mutations in *PARK2* account for 50% of autosomal recessive juvenile Parkinsonism and 15% of sporadic PD cases with onset before 45 years of age. The structural variations of *PARK2* alternatively spliced transcripts are implicated in the mechanisms of juvenile Parkinsonism. *PARK2* transcripts without exons 3-5 or exons 2-7 have been detected to be increased in PD, and an alternatively spliced variant of parkin that lacks exon 4, which leads to null enzymatic activity, is upregulated in sporadic PD [94].

### AD

AD is the most common neurodegenerative disease, characterised by progressive impairment in cognitive function and behaviour. Environmental exposure, aging and gene mutations are suggested to play a synergistic role in the pathogenesis of AD. To date, more than 50 loci have been implicated in AD, although the functions and underlying disease mechanisms for most of those genes are still undetermined. Several genes and pathways are implicated in AD, including the Aβ cascade, tau, inflammation, and cholinergic and oxidative stress [95]. Some of the gene products can be found in the extracellular amyloid plaques and intra-neuronal neurofibrillary tangles in the brains of AD patients, which are hallmark histopathologies of AD.

Tau is encoded by the microtubule associated protein tau (*MAPT*) gene, which consists of 16 exons. Alternative splicing of exon 10 gives rise to two tau isoforms, 3R tau (exon 10 exclusion) and 4R tau (exon 10 inclusion) [96]. Moreover, the disrupted ratio between the 3R and 4R isoforms is involved in tauopathies and AD pathogenesis [97], as the 4R tau has been shown to have stronger activity in promoting microtubule assembly and lead to greater neurodegeneration than the 3R tau [98]. Several features including the weak 5’ and 3’ splice sites in *MAPT* exon 10, and the self-complementary stem loop at the 3’-end of exon 10 and the 5’-end of intron 10 can cause a relatively low level of exon 10 inclusion [99]. Mutations including IVS10+16 C>T that disrupts the stem loop structure (Fig. 3b) increase the binding of U1 small nuclear RNA and enhance *MAPT* exon 10 splicing, leading to the predominance of 4R tau in familial AD patients [100].

Misprocessing and accumulation of the Aβ protein, a proteolytic product of amyloid precursor protein encoded by the APP gene, is another hypothesis for AD pathogenesis [101]. There are two major isoforms of Aβ, Aβ40 and Aβ42, depending on the cleavage site of γ-secretase. Aβ42 is prone to aggregate and is the major component of amyloid plaques [102]. There have been about 60 mutations reported for APP and most of the pathogenic mutations are clustered in exons 16 and 17 that encode the cleavage sites for β- and γ-secretase [103]. *APP* is alternatively spliced into as many as 11 different mRNA transcripts. Alternative inclusion of exons 7 and/or 8 generates three major *APP* transcripts: *APP770* that contains both exons 7 and 8; *APP751* that lacks exon 8; and *APP695* that lacks both exons 7 and 8 [104]. Although APP695 is the predominant isoform in neurons, the other two minor isoforms are also suggested to be involved in AD, albeit to a lesser extent.

Presenilin-1, encoded by the *PSEN1* gene, is one of the core components of the γ-secretase complex that is responsible for the cleavage of APP and the generation of amyloid peptides [105–107]. Although most *PSEN1* mutations are reported as missense variations, several pathogenic mutations can affect the alternative splicing, especially those near recognised canonical splice sites [108]. For example, the A>G mutation in the acceptor splice site of intron 8 causes the skipping of exon 9, resulting in decreased Aβ40 production, increased Aβ42/Aβ40 ratio and disrupted cellular functions [109].

Presenilin-2 is another component of γ-secretase and is encoded by *PSEN2*. *PSEN2* has also been shown to harbour mutations affecting the alternative splicing. A one-base-pair deletion in *PSEN2* (c.1073-2delA) causes the loss of the canonical exon 12 acceptor site, resulting in exon 12 skipping and ultimately causes a frame-shift and premature termination codon [110]. The deletion of GA (c.342_343delGA) in *PSEN2* exon 5 [111] has been found to result in a partial intron 5 retention and create an alternatively spliced *PSEN2* transcript lacking exon 6 [112]. Although there are limited studies on exon 6 deletion in *PSEN2* transcript, this mutation has been implicated in the pathogenic mechanisms of sporadic AD, including increasing γ-secretase activity, repressing the unfolded protein response and regulating inflammatory responses to hypoxic stress [113, 114].

For the majority of sporadic AD patients, the presence of the ε4 allele of apolipoprotein E (*APOE*) is one of the primary genetic risk factors. *APOE* has three different allelic variants ε2, ε3 and ε4, where the presence of ε2 lowers the AD risk, while conversely the increased expression of ε4 increases the AD risk [115]. Although the mechanism of how *APOE* modifies AD risk is not completely understood, an additional copy of *APOE* ε4 is more likely to promote Aβ aggregation and is thought to increase the stability of Aβ oligomers when compared to *APOE* ε2 or *APOE* ε3 [52, 116–119].

### Amyotrophic lateral sclerosis (ALS) and frontotemporal dementia (FTD)

ALS is a progressive and fatal neurodegenerative disease featured by selective loss of both upper and lower motor neurons [120]. FTD is a common type of dementia in people under 65 years of age and may occur in combination with ALS. Although ALS and FTD differ in some clinical symptoms and neuropathological changes, they are recognised to form a broad neurodegenerative continuum [121]. It is now clear that the molecular genetics of ALS and FTD also overlap significantly, involving over-expression of TAR DNA-binding protein (*TARDBP*), *FUS*, *hnRNPA1*, Coiled-Coil-Helix-Coiled-Coil-Helix Domain Containing 10 (*CHCHD10*), and most importantly, the chromosome 9 open reading frame 72 (*C9ORF72*) gene [122]. The hexanucleotide G4C2 repeat expansion in the first intron or promoter region of *C9ORF72* is now known to be the most common genetic cause for ALS and FTD. The main disease mechanisms are typically split into three mechanisms: gain-of-function due to the toxic dipeptide-repeat proteins produced by non-AUG-initiated translation, gain-of-function from the accumulation of sense and antisense hexanucleotide G4C2 in RNA, and loss-of-function of *C9ORF72* through haploinsufficiency [123]. The RNA and dipeptide repeats form insoluble foci in multiple regions within the brain and often co-localise with various RNA-binding proteins [124]. Alternative selection of transcription start and termination sites gives rise to three *C9ORF72* RNA transcripts, leading to three protein variants [125]. Aberrant splicing of the expanded *C9ORF72* transcript may contribute to its cytotoxicity; however, the expansions have also been shown to form RNA G-quadruplex inclusions and sequester splicing factor hnRNP H to disrupt splicing in ALS brains [126].

Another ALS- and FTD-related gene that regulates RNA splicing of hnRNPs is the *TARDBP* gene, which encodes the TAR DNA-binding protein 43 (TDP-43). Pathogenic mutations in *TARDBP* compromise the function of TDP-43, interfere with *hnRNPA1* pre-mRNA splicing and result in inclusion of exon7B and accumulation of the cytotoxic longer form of hnRNP A1B [127]. In addition to the aforementioned causative genes for ALS and FTD, a large number of splicing defects in other genes such as the senataxin (*SETX*) and the optineurin (*OPTN*) genes have also been reported to contribute to disease phenotypes [128–131].

SMA is the leading genetic cause for infant death before the age of 2 years. Unlike other neurodegenerative disorders, SMA is a monogenic disease most commonly caused by deletion of the entire *SMN1* gene, which encodes the full-length survival motor neuron (SMN) protein [132]. Humans carry one or more copies of *SMN2*, which is identified as a duplicated unprocessed pseudogene that could potentially be translated into an identical protein to SMN. However, the synonymous T>C substitution in *SMN2* exon 7 alters an exonic splicing enhancer into an exonic splicing silencer, which predominantly leads to an unstable transcript missing exon 7. Nevertheless, with an increase in *SMN2* copy number, small but significant amounts of full-length transcript can be generated and its translation into normal SMN may result in a milder SMA phenotype in some cases [133].

### Familial dysautonomia (FD)

FD or Riley-Day syndrome is a rare genetic neurodegenerative disorder characterised by poor development and progressive degeneration of autonomic and sensory neurons. This disease is almost exclusively found in the Ashkenazi Jewish population [134]. Although non-Jewish cases have been rarely reported, the major haplotype mutation associated with FD is a single point mutation in intron 20 of the inhibitor of kappa light polypeptide (*IKBKAP*) gene: IVS20+6 T>C [135]. This mutation weakens the 5’ splice site in *IKBKAP* intron 20 and results in a frameshift caused by skipping of exon 20. Skipping of the out-of-frame exon 20 results in a premature termination codon in exon 21, inducing nonsense-mediated decay of the *IKBKAP* transcript [136]. As IKBKAP is involved in the development and survival of peripheral neurons, depletion of this protein results in progressive degeneration of autonomic and sensory neurons [137].

### Expansion diseases

To date, more than 40 diseases have been linked to expansions of microsatellites at various intragenic regions, leading to various mechanisms of disease [138–141]. The most common mechanism in neurodegenerative expansion diseases is the toxic gain-of-function, leading to protein misfolding and insoluble protein aggregation, a hallmark of neurogenerative diseases [8]. Although protein misfolding is the most common phenotypic event, aberrant splicing has been reported in several expansion diseases such as Huntington’s disease and the spinocerebellar ataxias. These events have been excellently reviewed in [142, 143], and although not the focus of the review, it is important to highlight the wide range involvement of aberrant splicing in diseases.

## Antisense oligonucleotide (AO)-mediated splice-switching strategies for neurodegenerative diseases

AOs are single-stranded synthetic nucleic acid analogues that are usually 12–30 nucleotides in length and can be designed to specifically bind to target sequences through Watson-Crick base pairing. AOs can be used to manipulate gene expression through a variety of mechanisms including inducing mRNA decay, modulating splicing, masking microRNA-binding, blocking/increasing translation, etc. The mechanisms of AOs have been recently reviewed [144]. These mechanisms are achieved by targeting various *cis-*acting gene regulation elements and are typically dependent on their backbone chemistries and base modifications. For example, gapmers that contain a central block of deoxynucleotides flanked by blocks of 2’-*O*-methyl modified ribonucleotides can induce RNase-H to degrade target mRNAs; whereas fully modified peptide nucleic acids or phosphorodiamidate morpholinos (PMOs) are more suited for use as steric blockers or sterically blocking motifs involved in splicing, protein translation or polyadenylation [145–148]. The main focus of this review is on AO-mediated splicing-switching strategies for neurodegenerative disorders, thus we will not expand on the development of AO chemistries/backbone modifications. Chemical evolution of AOs, its relationship to the mechanisms of AO action and AO delivery methods have been discussed in a recent review [149].

**Fig. 4:**
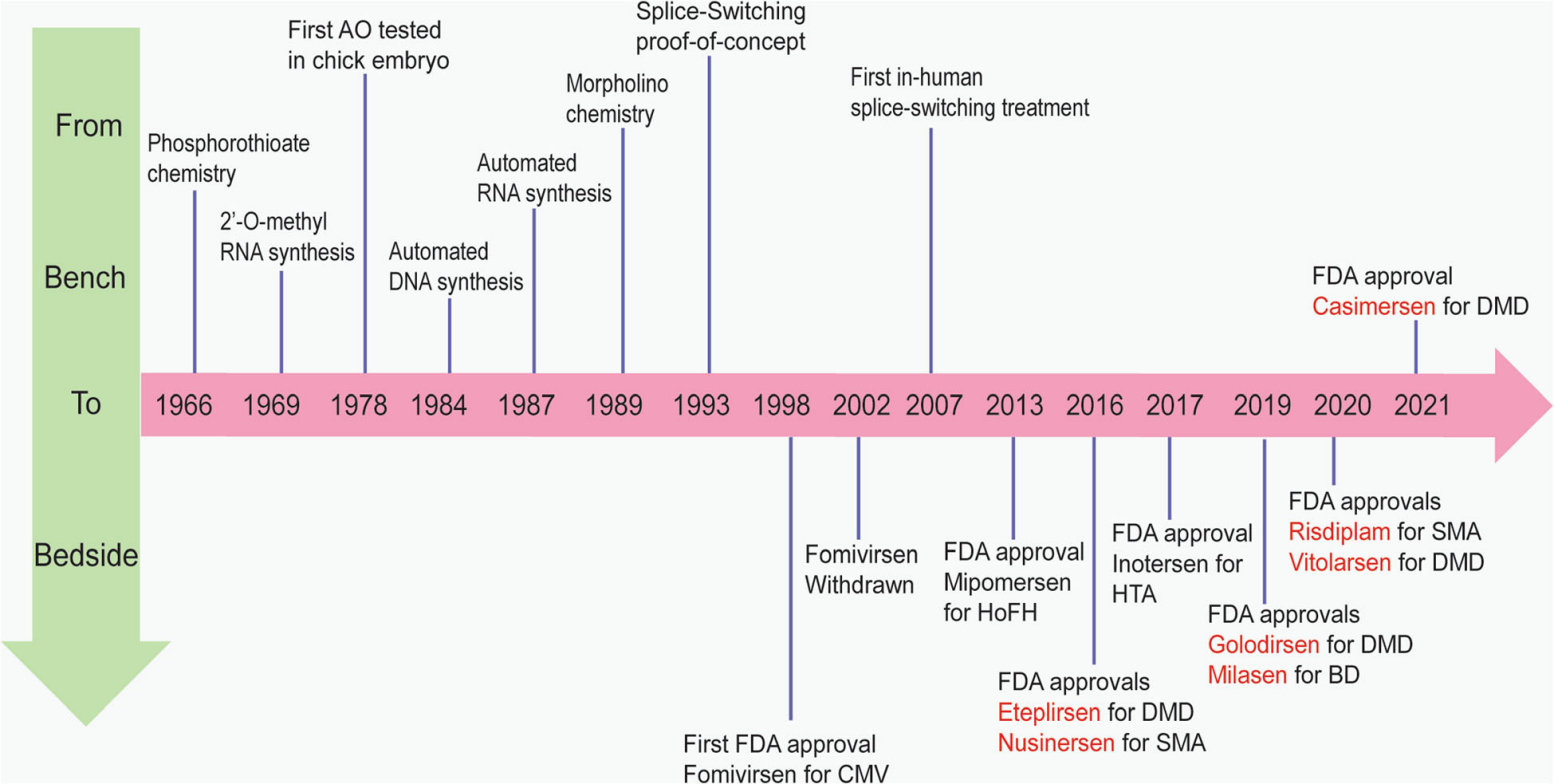
Milestones of the development of antisense oligonucleotide therapeutics (excluding siRNA) from bench to bedside. Approved drugs in red are splice-switching antisense oligomers. AO: antisense oligonucleotides; FDA: US Food and Drug Administration; CMV: cytomegalovirus retinitis (in immunocompromised patients); HoFH: Homozygous familial hypercholesterolemia; DMD: Duchenne muscular dystrophy; SMA: spinal muscular atrophy; HTA: Hereditary transthyretin-mediated amyloidosis; BD: Batten disease.

AO modification of gene expression was first reported in the study by Zamecnik and Stephenson, in which the ribosomal RNA translation of Rous sarcoma virus was inhibited by a complementary 13-nucleotide DNA molecule *in vitro* [150], presumably through the induction of RNase-H to degrade the mRNA. Since then, other RNase-H-inducing AOs including Fomivirsen, Mipomersen and Inotersen have been developed and approved by the US Food and Drug Administration (FDA) for the treatment of inherited and acquired diseases. A schematic of the major milestones in AO drug development and approvals (excluding small interfering RNAs) is shown in Fig. 4. Considerable experience has been gained in the development of splice-switching AOs in the past decade (Fig. 4), and the majority of AOs that have been approved by the FDA are designed to specifically modify the pre-mRNA processing.

### Splice-switching AOs

**Fig. 5:**
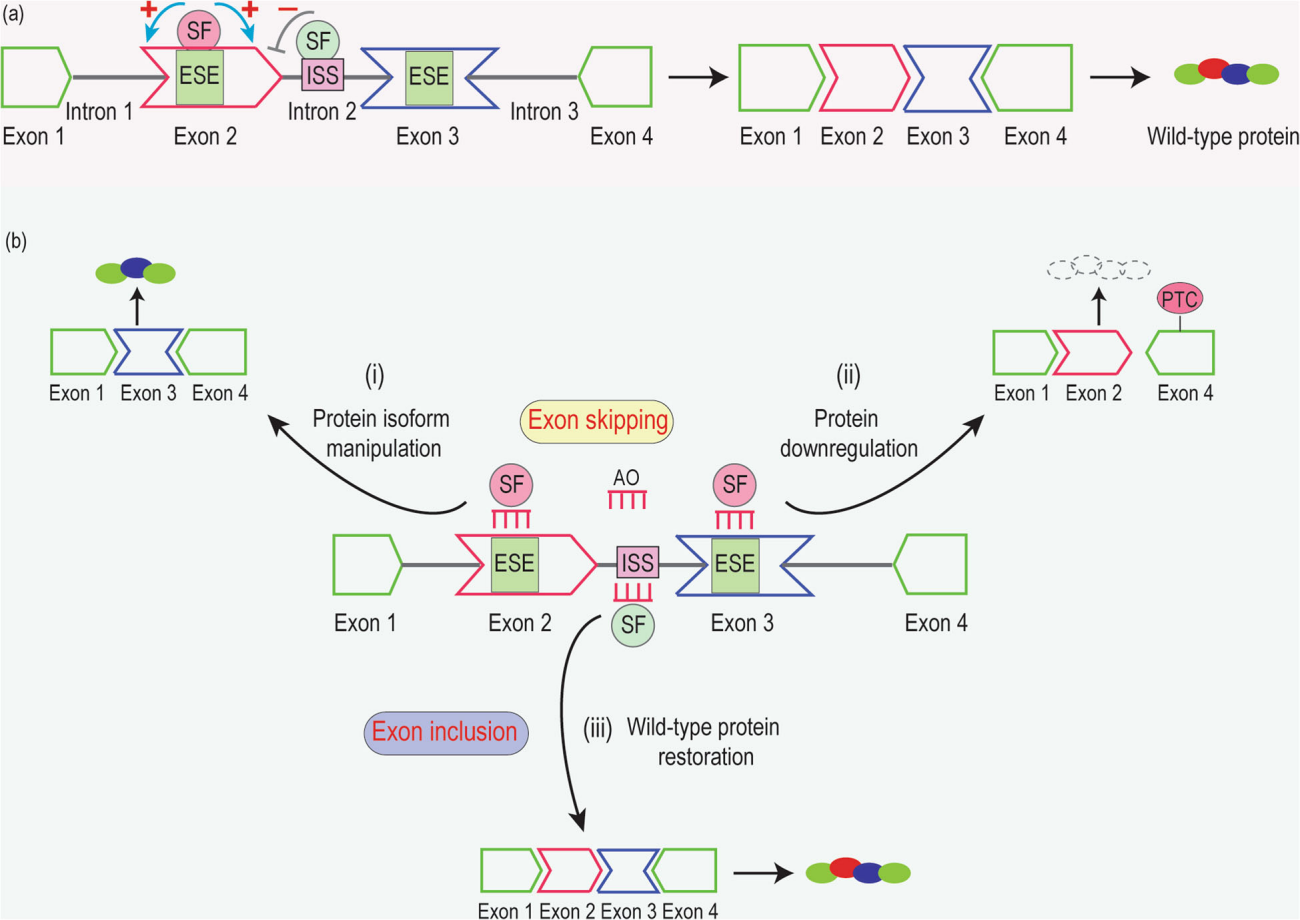
Mechanisms of action of splice-switching antisense oligonucleotides. **a** Stimulating splicing factors (SF) shown in pink circles such as SR proteins binding to exon splicing enhancers (ESE) promote the inclusion of an exon, while inhibitory SF in green circles such as hnRNPs binding to intron splicing silencers (ISS) inhibit exon inclusion. When promoting outweighs inhibiting actions, exons are included to generate a full-length transcript and wild-type protein. **b** Antisense oligomers (AOs) annealing to ESE blocks the interaction between SF and ESE and induces targeted (i) in-frame exon skipping, thus inducing in-frame transcripts and correspondingly new protein isoforms; and (ii) out-of-frame exon skipping and disrupts the reading frame and creates premature stop codon (PTC) in a downstream exon, that may lead to nonsense-mediated mRNA decay of the targeted transcript and downregulation of the protein. (iii) AOs anneal to ISS to increase targeted exon inclusion and generate a full-length transcript and wild-type protein.

With the wide recognition of the significance of pre-mRNA splicing in disease pathology, there is a need to understand this process and the ability to manipulate mRNAs for therapeutic outcomes. Splice-switching AOs can be designed to anneal across splice motifs, including exon splicing acceptor/donor sites and/or exon splicing enhancers, to block the interactions between these *cis-*acting elements and *trans-*acting proteins, thus interfering with pre-mRNA processing (Fig. 5). Although *in silico* prediction programs can sometimes be of value in designing splice-switching AOs, in our experience an empirical approach is most reliable as described [151]. Nevertheless, no splice motifs have emerged as consistent and reliable AO targets for efficient AO-mediated splice-switching as shown by previous studies [152–155]. In addition, AO design including the length, base composition and secondary structure has been shown to greatly influence the activity of splice-switching AOs.

Particular AO sequence motifs are also identified to affect AO activities and cause AO-related toxicities, including stimulating proinflammatory and immune responses. A sequence motif analysis revealed that specific GU-rich 4-mer motifs such as UUGU, GUUC, UGUU and UCUC can activate human Toll-like receptors through inducing the release of proinflammatory cytokines and chemokines from human peripheral blood monocytes [156]. Other factors influencing AO design include the presence, position and number of unmethylated CpG motifs in single-stranded DNA molecules, as they can also bring unwanted effects in addition to the non-specific binding to serum proteins. Polyanionic and negatively charged phosphorothioate AOs are known to bind to proteins, including intracellular and extracellular receptors that can lead to renal and/or hepatic toxicity. Certain splice-switching AO chemistries, such as PMOs, have been shown to elicit little or no off-target effects in long term, both *in vitro* and *in vivo* [157–161].

Although AO chemistries and toxicities have been bottlenecks for AO drug development, recent advances in oligo synthesis (in chemistries, scale and cost of production) have begun to address this hurdle in AO therapeutics (Fig. 4). In recent years, the development of splice-switching therapeutics for some neurodegenerative diseases has been extraordinary. Within the last five years, six splice-switching AO molecules have been approved by the US FDA, four of which are for the treatment of Duchenne muscular dystrophy (DMD). Eteplirsen (Sarepta Therapeutics), a 30-nucleotide PMO, is designed to skip DMD exon 51, Casimersen (Sarepta Therapeutics), is designed to skip DMD exon 45, and Golodirsen (Sarepta Therapeutics) and Viltolarsen (NS Pharma) are designed to skip DMD exon 53 [162–164]. These four drugs now address around 30% of all DMD mutations. The development of these AO drugs is based on the genotype-phenotype correlations that some exons are not essential regarding the functionality of the dystrophin protein. Although there is heterogeneity in patients with Becker muscular dystrophy (BMD), BMD patients with in-frame deletions in the central rod domain of the dystrophin protein often manifest with milder symptoms compared to DMD caused by out-of-frame deletions [165]. Therefore, antisense compounds are designed to block splice enhancers, thus the recognition of targeted DMD exons by spliceosome. Excising exons that flank the DMD-causing out-of-frame exons restores the reading frame and generates a semi-functional, truncated dystrophin protein as a disease-modifying treatment for DMD.

In addition to the FDA-approved antisense drugs for DMD, Nusinersen, a splice-modulating AO designed to specifically bind to a splicing silencer motif in exon 7 of *SMN2*, promotes the inclusion of exon 7 and the production of the full-length SMN protein [166]. The T>C substitution in *SMN2* creates an exon-splicing silencer and leads to the omission of exon 7 and an unstable SMN protein that is subject to rapid ubiquitin-proteasome degradation. By binding to the splicing silencer, Nusinersen blocks the negative elements recognised by *trans-*acting splicing factors including hnRNPs and inhibits the “looping-out” of *SMN2* exon 7 [167], thus producing a full-length, functional SMN protein. Patients with SMA who received intrathecal injections of Nusinersen showed improvements in motor function and required no ventilation assistance, when compared to the placebo cohort in a clinical trial [168]. The positive results from clinical trials then led to the approval of the drug for the treatment of SMA by the US FDA, European Medicines Agency and various medicine administrations in other countries, including China.

Another example showing the rapid development of splice-switching therapies for neurological conditions is the FDA approval of Milasen in 2019. Milasen was approved by the US FDA less than one year after the first contact between scientists and a single patient suffering from Batten’s disease [169, 170]. The approval of this “N-of-1” study may lead to regulatory changes and encourage a paradigm shift for small-cohort clinical trial design. If a new clinical trial model is established, it would bring huge benefits for the development of AO-mediated precision medicine for neurodegenerative disorders. For example, splice-switching strategies targeting one exon of one PD-causing gene will require patients participating in clinical trials to be stratified according to the genetic background, making the target patient cohort very small. Novel regulatory paradigms would, to some extent, facilitate the evaluation of potential splice-switching therapies for neurodegenerative diseases.

### Potential splice-switching therapeutics for PD

With the mounting evidence of aberrant splicing in PD pathogenesis, recent studies are utilising AOs to correct causative splicing defects in PD patients. Splice-switching AOs have been designed to induce skipping of *LRRK2* exon 2, leading to the generation of a premature stop codon in the transcript. With this strategy, *LRRK2* transcript and protein levels are decreased by approximately 50% and mitophagy function restored in PD patient fibroblasts carrying the *LRRK2* G2019S mutation [171]. Another approach that has been tried to reduce LRRK2 protein is the removal of exon 41. Although only moderate skipping of *LRRK2* exon 41 and LRRK2 protein reduction are achieved *in vitro*, improved calcium homeostasis has been demonstrated in patient iPSC-derived neurons with *LRRK2* G2019S mutation [172]. Subsequently, a single intracerebroventricular injection of AO has been shown to induce efficient *LRRK2* exon 41 skipping and reduced LRRK2 kinase activity in human *LRRK2* transgenic mice [173]. The AO-mediated LRRK2 downregulation strategy is now under a phase I clinical trial as a potential therapeutic approach for *LRRK2*-related PD [157].

Located within a chromosomal fragile site, genomic deletions are responsible for half of all *PARK2* mutations. Clinical genotype-phenotype studies have shown that PD patients carrying the out-of-frame genomic deletions of *PARK2* exon 3 or 4 have more severe symptoms and an earlier disease onset than patients harbouring the in-frame genomic deletion of both exons 3 and 4 [174, 175]. In addition, studies mapping the functional domains of the parkin protein have demonstrated that deleting the ubiquitin-like domain and the linker region encoded by *PARK2* exons 3 and 4 does not compromise the parkin catalytic activity [176]. These genotype-phenotype correlations justify an approach to excise one of these exons as a potential treatment for patients carrying amenable mutations. Splice-switching AOs targeting the splicing motifs of *PARK2* exon 4 have been shown to induce exon 4 skipping and restore functional parkin expression in fibroblasts derived from a PD patient carrying a heterozygous exon 3 deletion [177]. The induced shorter parkin protein can function to maintain mitochondrial homeostasis and transcriptionally repress *p53* expression [177]. Although further investigations are needed to prove the efficacy of this approach, this strategy may provide new avenues for AO-mediated treatment of PD.

α-Synuclein is another potential target for the development of disease-modifying therapies for synucleinopathies. Manipulating *SNCA* isoforms with splice-switching AOs could be an alternative option for PD treatment, since isoforms including SNCA126 and SNCA41 are less likely to form toxic α-synuclein aggregates [77, 83]. α-Synuclein pathology has been found to accumulate in anterior olfactory nuclei years prior to the development of motor symptoms [178]. This suggests that switching *SNCA* isoforms might have to be performed in the prodromal stage of PD to reduce the risk of developing motor symptoms, which poses considerable additional challenges, cost and duration of clinical evaluation.

### Potential splice-switching strategies for AD

Accumulating evidence has supported the central role of APP and Aβ in the development of AD, therefore efforts such as AO-mediated modulation of Aβ, especially Aβ42 expression, are currently under investigation as potential AD treatments [179]. Different antisense strategies targeting *APP* mRNA have been shown to reduce the APP protein to 39%–82% of normal levels and improve the cognitive functions in a mouse model of AD [180]. Since the exon 17 of *APP* encodes the γ-secretase cleavage site, which generates Aβ42, removing this cleavage site is hypothesised to reduce toxic Aβ42 expression and aggregation. In a recent study, treatment with AOs targeting *APP* exon 17 splicing motifs resulted in an *APP* transcript lacking exon 17, leading to reduced Aβ42 both *in vitro* and *in vivo* [181]. Another gene shown to be upregulated in AD patient brains is *BACE1*. A study has demonstrated that AOs designed to skip the out-of-frame *BACE1* exons can reduce BACE1 expression [182]. However, this study is preliminary, and the AOs used are still in the early stage of development, so further studies are needed to demonstrate the long-term consequences of these novel *BACE1*-targeting AOs as therapeutic strategies.

Of the three major ApoE isoforms ApoE2, ApoE3 and ApoE4 [183], the E4 isoform is strongly associated with the onset of AD and disease progression, thus reducing the ApoE4 level is hypothesized to induce reduction of Aβ accumulation and attenuation of cognitive deficits. An antisense approach has been investigated in an attempt to downregulate the disease-susceptible ApoE4 isoform in neonatal mice, resulting in a significant reduction of the initiation of Aβ accumulation and Aβ plaque size [184]. Although the exact mechanisms of how ApoE4 affects Aβ metabolism and increases AD risk remain to be determined, the ApoE receptor 2 (*ApoER2*) appears to mediate the pathological synergistic interactions between ApoE4 and Aβ [117]. Since dysregulated splicing of *ApoER2* exon 19 has been observed in brain samples from AD patients, the splice-switching AO strategies have been used to enhance exon 19 skipping and have been shown to improve synaptic function and memory in an AD mouse model [185].

Another approach has been to target tau expression levels. A splice-switching strategy aiming to excise *MAPT* exon 10 and thereby convert 4R tau to 3R tau offers an alternative strategy to alleviate the tauopathy. This splice-switching approach is likely to be less toxic as it would only shift the relative ratio of the two isoforms to confer a more protective effect, rather than complete downregulation of all isoforms.

### Splice-switching approaches for other neurodegenerative disorders

Several pathogenic mechanisms of expanded *C9ORF72* have been implicated in ALS and FTD diseases, hence reducing the repeat expansions is being considered as a potential treatment for patients. In addition to the allele-specific knockdown of the expanded *C9ORF72* allele [186], reducing *C9ORF72* expression using splice-switching AOs to skip out-of-frame exons similar to that depicted in Fig. 5b(ii) could also downregulate *C9ORF72*. However, reducing the levels of the non-expanded transcripts could lead to autophagy deficits [187]. Since the disease-associated repeat expansion is only present in the *C9ORF72* transcript starting with exon 1a [125], altering the *C9ORF72* transcription start site could be another possible approach as indicated by a recent study showing AO induction of transcriptional blocking [188]. Similar strategies can also be considered for patients with other microsatellite repeat expansion disorders including spinocerebellar ataxia type 3 (SCA3), Huntington’s disease, spinal bulbar muscular atrophy and fragile X syndrome, although the location, length and the repeating units of these microsatellite repeat expansions may vary [189]. For example, the CAG repeat expansion is located in *ATXN3* exon 10 (the causative gene for SCA3); and various splice-switching AOs have been tried to remove the repeat expansion containing exon 10 and reduce the polyglutamine-expanded Ataxin-3 protein both *in vitro* and *in vivo* [155, 190].

Recently, two ALS patients who received intrathecal administration of an adeno-associated virus encoding a microRNA targeting the superoxide dismutase type 1 (*SOD1*) gene had transient improvement in leg strength and a stable vital capacity during a 12-month follow-up period [191], suggesting the therapeutic benefits of downregulating SOD1. The splice-switching strategy (Fig. 5b(ii)) based on an FDA-approved chemistry is an alternative approach to knockdown SOD1. By skipping an out-of-frame *SOD1* exon, a different *SOD1* transcript isoform is generated with a premature stop codon, which is subjected to nonsense-mediated decay and thus decreasing SOD1 protein expression [192].

Since most neurodegenerative disorders have highly complicated aetiologies and relatively slow pathogenesis where mutations in multiple genes are involved, splice-switching AOs targeting one gene or one mRNA isoform are likely to be applicable to only a certain proportion of patients with these diseases. For example, mutations in *FUS* make up only 2.8%–6.4% of familial ALS cases (familial cases only account for 10% of all ALS patients), thus correcting these mutations by AOs would only address a small ALS population, creating challenges for clinical trial design. However, since the FDA approval of the “N-of-1” study for Batten’s disease, regulatory changes have been made, increasing the likelihood that personalised medicine may become available for individuals or small populations with rare diseases and highly amenable mutations.

## Conclusions

AOs, especially splice-switching AOs, have the capacity and potential to reduce, restore or manipulate the expression of mRNAs and their translated proteins with high specificity. Thus, they can be used to target a variety of diseases, in particular neurodegenerative diseases where abnormal, or inappropriate splicing defects are especially common. The delivery of AOs to the central nervous system is further improving with the advancement in AO chemical modifications and delivery carriers which include cell-penetrating peptides and polymer-based nanoparticles. Unlike the viral vector-mediated siRNA approaches or gene therapy, regular AO administrations are needed to maintain long-term therapeutic benefits, and this comes with both advantages and disadvantages. The application of these AOs does not constitute gene therapy in the usual sense as the genome of the patient is not modified, but gene expression is specifically altered. Although re-administration is required, AO delivery can be readily withdrawn if adverse effects are encountered, or a more effective treatment becomes available. For example, DMD individuals receiving weekly infusions of Eteplirsen, Golodirsen, Viltolarsen or Casimersen could easily transfer across to one of the viral gene replacement therapies upon validation of the safety and efficacy of a therapy that is not restricted to a specific subset of mutations. Unfortunately, a recent clinical trial update failed to show the efficacy of one gene therapy for DMD patients. The development of most AO-mediated splice-switching approaches is at a very early stage and it has the ability to change the landscape of precision medicine for neurodegenerative disorders. Although huge efforts are needed to overcome the challenges ahead, including animal modelling for preclinical studies and clinical trial design for subsets of patients when personalised medicine is considered, the emerging splice-switching therapeutics could be a game changer in the development of disease-modifying treatments for neurodegenerative disorders.

## Data Availability

Not applicable.

## Abbreviations

Aβ: Amyloid-β
AD: Alzheimer’s disease
ALS: Amyotrophic lateral sclerosis
AO: Antisense oligonucleotides
DMD: Duchenne muscular dystrophy
FD: Familial dysautonomia
FDA: Food and Drug Administration
FTD: Frontotemporal dementia
ISS: Intron splicing silencers
PD: Parkinson’s disease
PMO: Phosphorodiamidate morpholino oligomers
PTC: Premature stop codon
SCA: Spinocerebellar ataxia
SMA: Spinal muscular atrophy
SMN: Survival motor neuron
SNP: Single nucleotide polymorphisms
snRNA: Small nuclear RNAs
snRNPs: Small nuclear ribonucleoproteins
tKO: Triple knockout
UTR: Untranslated region
