# Correction to: Neurodegenerative diseases: a hotbed for splicing defects and the potential therapies

**DOI:** 10.1186/s40035-021-00267-w

**Published:** 2021-10-25

**Authors:** Dunhui Li, Craig Stewart McIntosh, Frank Louis Mastaglia, Steve Donald Wilton, May Thandar Aung-Htut

**Affiliations:** 1 Centre for Molecular Medicine and Innovative Therapeutics, Health Futures Institute, Murdoch University, Perth, Western Australia Australia; 2 Perron Institute for Neurological and Translational Science, Centre for Neuromuscular and Neurological Disorders, The University of Western Australia, Perth, Western Australia Australia

## Correction to: Translational Neurodegeneration (2021) 10:16 https://doi.org/10.1186/s40035-021-00240-7

Following publication of the original article [1], the authors would like to correct a formula from “T > C” to “C > T” in two paragraphs.

1. In the third paragraph of the section **Amyotrophic lateral sclerosis (ALS) and frontotemporal dementia (FTD)**, the correct sentence should be:

However, the synonymous C > T substitution in SMN2 exon 7 alters an exonic splicing enhancer into an exonic splicing silencer, which predominantly leads to an unstable transcript missing exon 7.

2. In the fourth paragraph of the section **Splice-switching AOs**, the correct sentence should be:

The C > T substitution in SMN2 creates an exon-splicing silencer and leads to the omission of exon 7 and an unstable SMN protein that is subject to rapid ubiquitinproteasome degradation.

In addition, the authors identified an error in Fig. 4. The correct figure is given below:

**Fig. 4:**
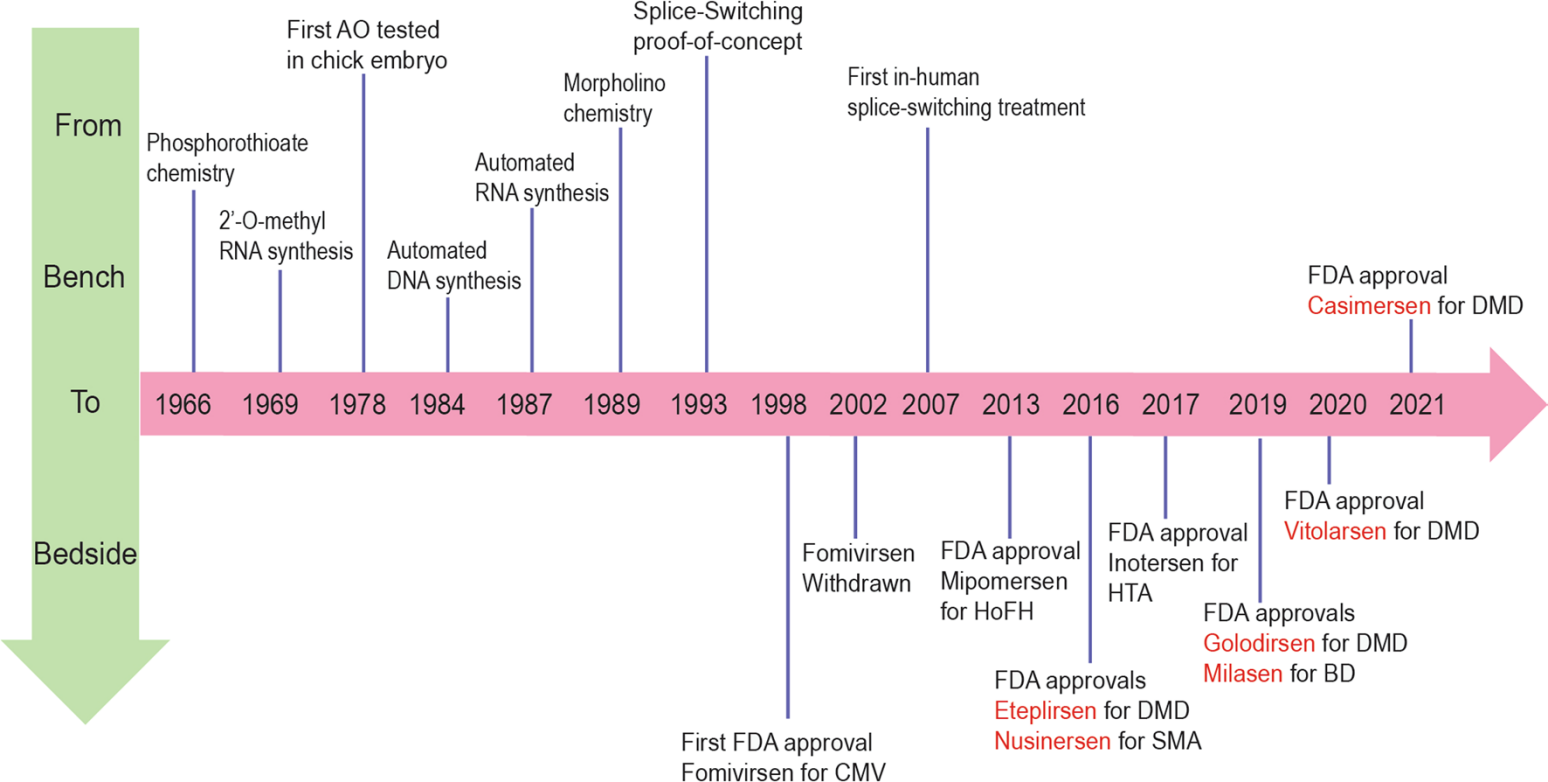
Milestones of the development of antisense oligonucleotide therapeutics (excluding siRNA) from bench to bedside. Approved drugs in red are splice-switching antisense oligomers. AO: antisense oligonucleotides; FDA: US Food and Drug Administration; CMV: cytomegalovirus retinitis (in immunocompromised patients); HoFH: Homozygous familial hypercholesterolemia; DMD: Duchenne muscular dystrophy; SMA: spinal muscular atrophy; HTA: Hereditary transthyretin-mediated amyloidosis; BD: Batten disease

The original article [1] has been corrected.
